# 2026 ETA guideline on interference in immunoassay measurements used in assessment of thyroid function

**DOI:** 10.1530/ETJ-26-0011

**Published:** 2026-08-14

**Authors:** Irene Campi, Ulla Feldt-Rasmussen, Damien Gruson, David Halsall, Veronique Raverot, Sjoerd van den Berg, Carla Moran

**Affiliations:** ^1^ Department of Endocrine and Metabolic Diseases, IRCCS Istituto Auxologico Italiano, Milan, Italy; ^2^ Department of Nephrology and Endocrinology, Copenhagen University Hospital, Rigshospitalet, Copenhagen, Denmark; ^3^ Department of Clinical Medicine, Faculty of Health and Medical Sciences, University of Copenhagen, Copenhagen, Denmark; ^4^ Department of Clinical Biochemistry, Cliniques Universitaires St-Luc, UC Louvain, Brussels, Belgium; ^5^ Department of Clinical Biochemistry, Addenbrooke’s Hospital, Cambridge University Hospitals NHS Foundation Trust, Cambridge, UK; ^6^ Service de Biochimie et Biologie Moléculaire, Laboratoire de Biologie Médicale Multi-Sites (LBMMS), Hospices Civils de Lyon, Lyon, France; ^7^ Department of Internal Medicine, Division of Endocrinology, Erasmus MC, University Medical Center Rotterdam, Rotterdam, Netherlands; ^8^ Department of Clinical Chemistry, Erasmus MC, University Medical Center Rotterdam, Rotterdam, Netherlands; ^9^ Endocrine Section, Beacon Hospital, Dublin, Ireland; ^10^ Endocrinology Department, St Vincent’s University Hospital, Dublin, Ireland; ^11^ UCD School of Medicine, Health Sciences Centre, Dublin, Ireland

**Keywords:** thyroid function tests, free T4, immunoassays, assay interference

## Abstract

Although measurement of thyroid hormones (THs), TSH and thyroid autoantibodies, is usually straightforward, erroneous results are occasionally generated, raising the possibility of incorrect or delayed diagnoses, unwarranted investigations, and provision of inappropriate treatments. Assay interference can be acquired (e.g. antibodies), genetic (e.g. variant TH-binding proteins), or pharmaceutical (e.g. displacing agents and biotin) in nature. In those with discordant thyroid function, assay interference must be excluded prior to the diagnosis of rare inherited (resistance to thyroid hormone) or tumoural conditions (TSHoma). Liaison between the clinician and clinical biochemistry teams is essential when assay interference is being investigated; appropriate further biochemical testing depends on the clinical picture, initial biochemistry, and local biochemical expertise. In a subset of cases, access to a regional centre where patients can be clinically assessed, in conjunction with performance of specialised clinical laboratory testing (biochemical, +/− genetic), may be required. When assay interference is identified, clear communication with the patient (ideally aided by reliable, accessible patient literature) and documentation to their physician are necessary. Professionals must stay updated on advances in testing and be alert for evolving assay interferences. An accompanying plain language summary is intended to provide an accessible overview of the key messages and recommendations contained in this guideline for non-specialist readers, including patients, carers, healthcare professionals outside the speciality, and the wider public.

## Methodology and grading

The executive committee of the European Thyroid Association (ETA), following consultation with its guidelines and publications committee, commissioned the development of this guideline, led by one chairperson (CM). The guideline task force, appointed by the chairperson and ETA guidelines and publications committee, consisted of three endocrinologists (IC, UFR, and CM) and four specialists in laboratory medicine (DJH, DG, VR, and SvdB). All panellists had clinical and/or clinical laboratory expertise in the investigation of discordant function tests. The manuscript and specific recommendations were developed by reviewing the best available research evidence, with the knowledge and technical experience of the panel. The Grading of Recommendations, Assessment, Development, and Evaluation (GRADE) framework was used for grading the quality of evidence and making recommendations (1, 2). The quality of the evidence was graded as level 1: high (RCT evidence/meta-analysis – high-quality evidence (ØØØØ)); level 2: moderate (intervention short of RCT or large observational studies – moderate quality (ØØØO)); level 3: low quality (case–control studies and case series – low quality (ØØOO)); and level 4: very low quality (case reports and expert opinion – very low-quality (ØOOO)) using the modified GRADE criteria (3). The strength of a recommendation was categorised as either strong (S; a recommendation) or weak (W; a suggestion, not a recommendation). The final draft of the manuscript was sent to the guidelines and publications committee for comments and thereafter posted on the ETA website for four weeks for feedback from the ETA members. All proposed changes and comments were taken into consideration, and the resulting revisions were incorporated into the final documents submitted to the European Thyroid Journal after approval by the guideline board.

## Introduction

Accurate measurement of thyroid hormones (THs), including thyroid-stimulating hormone (TSH), free and total fractions of triiodothyronine (T3) and thyroxine (T4), and testing for thyroid autoantibodies and cancer-associated biomarkers (thyroglobulin and calcitonin), is key for diagnosing and managing thyroid disorders. Immunoassays remain the standard in clinical laboratories because of their sensitivity, specificity, automation, and rapid turnaround (4). However, their reliability can be compromised by analytical interferences that may lead to diagnostic errors and inappropriate management (5).

Interference arises when endogenous or exogenous substances disrupt antibody–antigen binding or signal detection. Common sources include heterophilic antibodies, biotin, and autoantibodies directed against THs or assay components. Heterophilic antibodies may link capture and detection antibodies, generating spurious signals, while biotin (widely used as a supplement) has previously been described to cause falsely high or low hormone results in streptavidin–biotin-based assays (6).

These interferences can result in misclassification of thyroid status, underscoring the need for clear clinical guidelines and mitigation strategies, such as blocking reagents, assay redesign, or alternative technologies less prone to interference (6). Advances in immunoassay platforms, automated mass spectrometry, and AI-supported assay development may further enhance test robustness.

Here, we summarise the current understanding of immunoassay interference, outline approaches for detection and resolution, and offer guidance for clinicians and clinical laboratory professionals managing patients with confirmed or suspected assay interference in tests used to measure THs or related substances.

## General principles

Thyroid disease is common, affecting over 8% of the population (2.5% thyrotoxicosis; 5.8% hypothyroidism) (7). Although TH measurement is generally straightforward, interfering substances can cause inaccurate results, leading to misdiagnosis, inappropriate investigations, or unnecessary interventions. In a review of >150 cases, ≥50% of patients experienced negative clinical consequences from assay interference, whereas only 8% had no impact (6). Thus, interference is clinically significant and may cause patient harm if unrecognised.

People with discordant thyroid function tests (TFTs) due to assay interference or genuine causes may present in any clinical setting. Identification of assay interference as a cause of discordant TFTs can be challenging. Specialist laboratories will have expertise in investigation of discordant TFTs, while others will have limited capability to investigate such patients. We acknowledge that different clinics/laboratories have varying levels of expertise; some basic tests for interference may be done locally, while more specialised testing will require regional specialist services. It will not be possible or practical for all laboratories to develop assays for specialist investigation of thyroid function, so each clinical setting/laboratory should have access to a regional specialist centre where a biochemical workup can be performed and relayed back to the referring centre, and where patients can be referred for specialist clinical evaluation if indicated. We acknowledge that in resource-limited settings, access to such testing may be extremely limited.

Commercially available TSH and TH assays vary in susceptibility to interference, which may be assay-specific or genetically influenced (e.g. albumin or TSHβ variants). Assay architecture is continually updated to reduce interference, such as from biotin.

**Figure 1:**
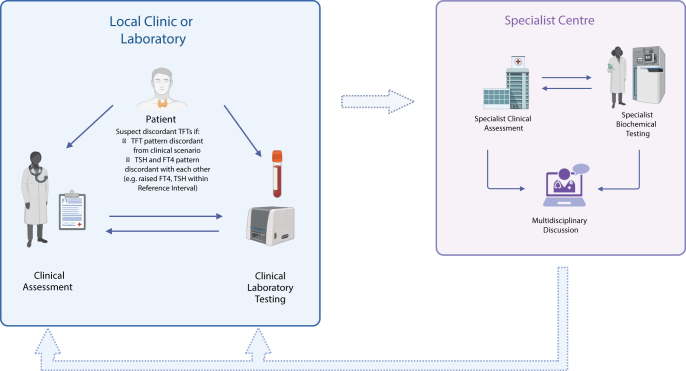
Algorithm demonstrating general approach to detection, investigation, and confirmation of assay interference. Assay interference may initially be suspected by a clinician or a clinical biochemist. In either instance, liaison between the clinician and clinical biochemist will ensure appropriate further testing. In some circumstances, specialist testing and/or clinical assessment may be required at a tertiary referral centre. Interdisciplinary discussion (specialists in laboratory medicine and clinicians +/− genetics), at either local or specialist centres, should occur in order to optimise testing and identification of interference. Created in BioRender by Moran C (2026) https://BioRender.com/uv0w3ur.

There are key steps required when assessing a patient with discordant TFTs, including i) careful history taking to note, in particular, thyroid symptoms, family history, and physiological states that affect THs (e.g. pregnancy and severe illness); ii) review of medications or supplements used (especially biotin and amiodarone); and iii) clinical examination for goitre and thyroid status (Fig. 1). Following the assessment, it may be possible to identify which assay (if any) seems erroneous. This is helpful information to the clinical biochemist.

Following assessment of the patient with discordant TFTs, liaison with the clinical biochemist is essential. Initial steps may include repeat testing using an alternate assay (preferably a two-step method) or dilution studies. Advanced methods, such as polyethylene glycol precipitation, may be needed, as outlined below.

If assay interference is identified, the findings should be clearly reported back to the patient and their referring physician. If an inherited cause of assay interference is identified (such as familial dysalbuminaemic hyperthyroxinaemia (FDH) or variant thyroxine-binding globulin (TBG)/transthyretin (TTR)/TSHβ subunit), screening should be offered to first-degree relatives. Clear, written information regarding the presence of assay interference should be provided to the patient, who can provide this to any treating physicians in future to avoid duplication of investigation or unnecessary treatment. It can be helpful to inform referring physicians which (if any) assays are reliable; if TSH assays are unaffected by assay interference, for example, the TSH concentration could be relied upon during a future assessment of thyroid status, if required. A summary of the task force recommendations is supplied in Table 1.

**Table 1:** Summary of recommendations.

**General considerations**	
1	We recommend that clinicians interpreting thyroid function tests and all clinical laboratory staff are aware of the possibility of assay interference in immunoassays. (ØØOO, S)
2	We recommend that endocrinologists and specialists in laboratory medicine be aware of how reference intervals are generated and how these are affected by physiological and pathological states and understand the limitations of TSH, FT4, and FT3 measurements. (ØØOO, S)
3	We recommend that endocrinologists and specialists in laboratory medicine know which type of assay was used to measure TSH, (F)T4, and (F)T3 in a particular patient and the limitations of these assays. (ØØOO, S)
4	We suggest that endocrinologists and specialists in laboratory medicine be aware of the general causes of assay interference in TSH and thyroid hormone measurements, such as antibody interference, thyroid hormone-binding protein abnormalities, anti-reagent antibodies, displacing agents, and others. (ØOOO, W)
5	We recommend that endocrinologists and clinical biochemists be aware that the susceptibility of assays to various forms of interference will change over time, with newer generation of assays being developed to overcome observed forms of interference. (ØØOO, S)
6	We recommend consideration of assay interference when thyroid function tests (TSH, (F)T4 +/− (F)T3) are incongruent with the clinical picture or with each other (e.g. elevated T4 but TSH within the reference interval). (ØØOO, S)
7	Following identification of a patient with suspected TSH, thyroid hormone, or thyroid autoantibody assay interference by a clinician, we recommend liaison with clinical laboratories to determine the most appropriate further biochemical test(s). (ØØOO, S)
8	If suspected TSH, thyroid hormone, or thyroid autoantibody assay interference is first identified by the specialist in laboratory medicine, we recommend liaison with the requesting clinician as the first step and thereafter performing further testing as clinically indicated. (ØOOO, S)
9	We recommend that all patients with suspected assay interference in TFTs have a clinical review, including medication history, family history, and clinical examination for signs of thyroid disease. (ØØOO, S)
10	We recommend that endocrinologists and clinical biochemists establish a communication pathway within their hospital/clinic to relay results of suspected assay interference and facilitate discussion of such cases. (ØØOO, S)
11	We acknowledge that availability of testing for assay interference locally will vary depending on the clinical laboratory expertise and may be limited. (ØØOO, S)
12	We recommend that all medical scientists and endocrinologists have access to a referral/specialist clinical laboratory where specialist testing for assay interference can be performed if indicated, such as method comparison, TSH dilution studies, immunosubtraction studies (typically with polyethylene glycol), gel filtration chromatography, equilibrium dialysis, testing for thyroid hormone-binding abnormalities, measurement of thyroid hormones by mass spectrometry, and others. (ØØOO, S)
13	We recommend that each local centre have access to a regional specialist centre where patients can be referred for specialist clinical evaluation if indicated. (ØØOO, S)
14	We recommend that centres offering specialised interference testing are adequately supported to perform ongoing specialist testing. (ØØOO, S)
15	We recommend ongoing research in development of new methods to identify and delineate causes of assay interference. (ØØOO, S)
16	Where assay interference is identified, we recommend that this information is formally communicated in writing to the primary care physician and the referring physician (if different to the primary care physician). The patient should also receive this information. (ØOOO, S)
17	If assay interference is identified, we suggest that clinical biochemists and endocrinologists provide advice to primary care physicians regarding measurements (TSH or FT4) that are reliable and could be used to aid assessment of thyroid status in future. This can be helpful in a patient with coexistent thyroid disease, or if symptoms suggestive of thyroid disease later arise. (ØOOO, W)
18	We suggest that accessible plain language information on assay interference, in written and/or online formats, be available to patients in whom assay interference is identified. (ØOOO, W)
19	Where an inherited cause of assay interference is identified (such as familial dysalbuminaemic hyperthyroxinaemia, TBG/transthyretin abnormalities, and TSHB subunit variant), we recommend that screening be offered to first-degree relatives who may be affected. (ØOOO, S)
**TSH assays**	
20	Where TSH assay interference is suspected, we recommend that further biochemical tests for TSH interference be performed. The method chosen will be dependent on the capabilities of the local clinical laboratory but may include TSH measurement in alternative assay(s) and TSH dilution studies. For some clinical laboratories, direct referral for more specialised testing at a referral laboratory at the outset may be most appropriate (Ab neutralisation, PEG precipitation, gel filtration chromatography; see Table 2). (ØØOO, S)
21	If TSH assay interference is confirmed, we suggest that the laboratory report contains information on the assay(s) affected and what tests have been performed to investigate the cause of the interference. Depending on the nature of the interference, the assessment of thyroid status is based on clinical status and FT4 measurement +/− an alternative assay for TSH. (ØOOO, W)
**T4/T3 assays**	
22	Where interference in FT4 and/or FT3 is suspected, we recommend further clinical laboratory testing, with the method chosen dependent on the capabilities of the local laboratory, such as FT4/FT3 measurement in alternative assay(s), total T4/total T3 measurement, and/or more specialised testing at a referral clinical laboratory. (ØØOO, S)
23	Where interference in FT4 and/or FT3 is suspected to be due to medications (e.g. biotin and displacing agents), we recommend repeat analyses of FT4 and/or FT3 after omission of the suspected agent, if possible. (ØØOO, S)
24	Where an inherited form of FT4 and/or FT3 assay interference is suspected (e.g. familial dysalbuminaemic hyperthyroxinaemia (FDH), transthyretin abnormalities (TTR), or TBG abnormalities (TBG)), we recommend familial TSH, FT4, and FT3 measurements. We recommend confirmation of diagnosis by genetic testing. (ØØOO, S)
25	If FT4/FT3 assay interference is confirmed, we suggest that the assessment of thyroid status is based on clinical status and TSH measurement. In some cases, quantification of true endogenous T4/T3 concentration may be made by measuring FT4/FT3 by an alternative assay/a two-step assay, following equilibrium dialysis, or TT4/TT3 by mass spectrometry. (ØOOO, W)
26	We recommend proceeding to specialist investigations for rare inherited or acquired causes of discordant TFTs (such as resistance to thyroid hormone b- or TSH-secreting pituitary tumours) *only after* assay interference has been definitively excluded by a specialist in laboratory medicine. (ØØOO, S)
**Other assays – thyroglobulin, ** **anti-** **TSH** ** receptor antibody, ** **anti-** **thyroglobulin** ** antibody, ** **anti-** **TPO** ** antibody, and calcitonin**	
27	We recommend that clinicians and clinical biochemists be aware of the possibility of thyroglobulin, thyroglobulin antibody, and calcitonin assay interference, which is especially relevant in the management of thyroid cancer patients. (ØØOO, S)
28	Although anti-TSH receptor antibodies have high sensitivity and specificity for Graves’ disease, we recommend that clinicians and specialists in laboratory medicine are aware of rare occurrences of assay interference in these assays, with the resultant possibility of misdiagnosis. (ØOOO, S)
29	We recommend that clinicians and clinical biochemists consider the possibility of a high-dose hook effect when unexpectedly low thyroglobulin or calcitonin concentrations are observed in patients with differentiated or medullary thyroid cancer, respectively, especially when results are particularly discordant with the clinical picture. (ØOOO, S)
**Future considerations**	
30	Assays for TSH, thyroid hormones and thyroid autoantibodies change over time, and we recommend that endocrinologists and specialists in laboratory medicine remain up to date in changes in assays and advances in measurement in order to implement these into their practice, as appropriate. (ØOOO, S)

## Fundamental principles of assay performance and interference

**Figure 2:**
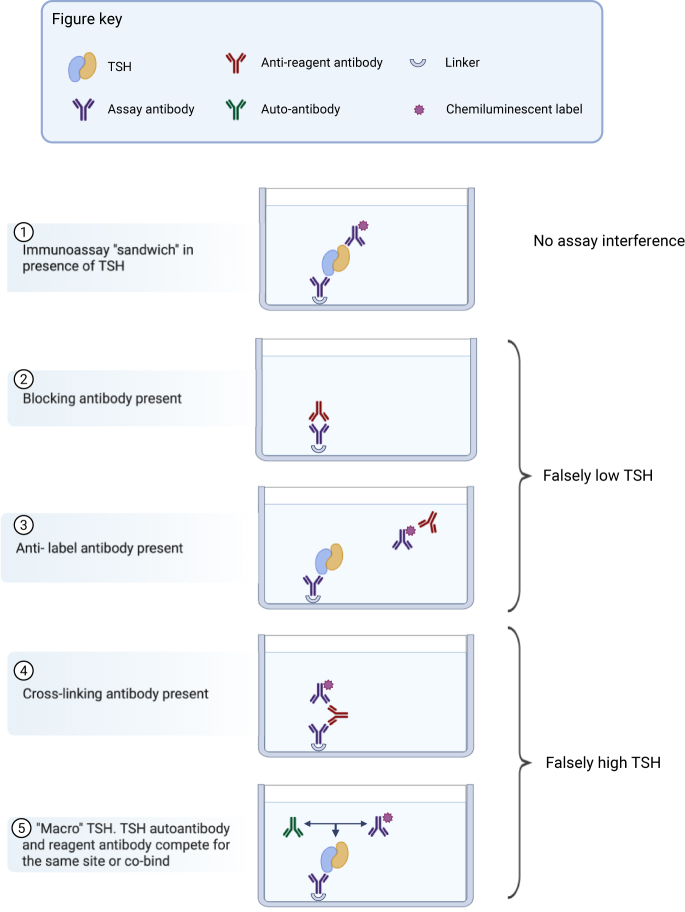
Mechanisms of antibody interference in TSH immunoassays. Schematic demonstrating mechanisms of assay interference in TSH immunometric immunoassays. A positive signal occurs when the chemiluminescent signal is immobilised to the solid phase; this occurs by sandwiching TSH between a capture and detection antibody (1). Falsely low TSH results (negative interference) can occur due to the presence of an antibody that binds directly to the capture antibody (2), or the labelled, detection, antibody (3). Falsely high TSH results (positive interference) occur when antibodies form a bridge between capture and detection antibodies (4), or endogenous TSH autoantibodies form immunoreactive but biologically inactive hormone complexes (macro-TSH) that are detected in the assay (5). Created in BioRender by Moran C (2026) https://BioRender.com/uv0w3ur.

Large molecules, such as TSH, can be measured using an immunometric (‘sandwich’) assay, where the TSH molecule is bound by a capture antibody (immobilised on a solid phase) and a detection antibody (the ‘tracer’, labelled with enzymes, fluorescence, radiation, or chemiluminescence) (Fig. 2). The signal generated is directly proportional to the TSH concentration (5). Assay interference occurs most commonly due to antibody interference, which can cause either falsely high or low TSH concentrations. Rarely, genetic variations in the TSH molecule can occur, which can cause falsely low TSH concentrations.

T4 and T3 molecules are smaller and more difficult to capture between two antibodies; thus, single-site immunoassays are typically used for thyroid hormone measurement. In this assay format, the concentration of hormones is estimated by competition between the hormone present in the sample and a labelled hormone or hormone analogue for available binding sites on an anti-T4/T3 antibody. Some assays use a labelled antibody and immobilised analogue. Thyroid hormones are also extensively protein bound with the small fraction of free hormone being biologically active. The single-site assay can be used for both total and free hormone assays depending on whether the protein-binding equilibrium is preserved (in free hormone assays) or disrupted (in total hormone assays). Unfortunately, preserving the *in vivo* equilibrium between free and bound hormone to enable accurate free hormone assessment is challenging, and any factor that disturbs this equilibrium may lead to assay interference (6).

**Figure 3:**
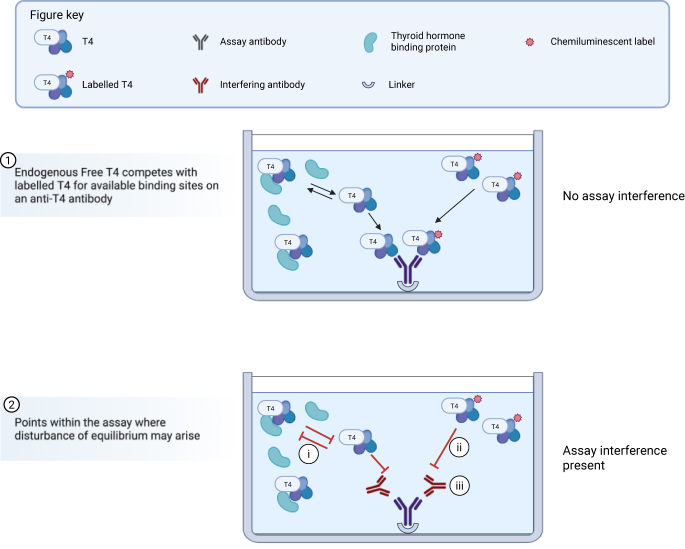
Mechanisms of interference in free T4 assays. Free T4 (FT4) concentrations are determined by indirect, competitive immunoassays. Labelled T4 and ‘free’ (endogenous) T4 compete for available anti-T4 antibody-binding sites; the more endogenous fT4 is present in the sample, the less labelled T4 is captured (1). The amount of T4 captured can be affected by (i) thyroid hormone-binding protein variants or displacing agents affecting the T4:FT4 equilibrium, (ii) endogenous anti-T4 autoantibodies binding the T4 tracer, or (iii) interfering antibodies blocking the capture antibody (2). FT4 assay interference is typically positive (i.e. less tracer is bound, and hence a falsely high FT4 result is generated), although weakly binding displacing agents can cause negative interference. Created in BioRender by Moran C (2026) https://BioRender.com/uv0w3ur.

The generated signal is inversely proportional to the concentration of T4/T3 present in the serum (5) (Fig. 3). Any factor that disturbs the *in vitro* equilibrium between the free THs (FT4/FT3) and thyroid hormone-binding proteins (THBPs) may lead to assay interference or shift the balance between bound and free TH fractions. This includes, for example, medications that displace THs from THBPs, or the presence of variant THBPs with altered affinity for THs or abnormal concentrations.

In addition, multiple forms of antibody interference can occur and may lead to elevated or decreased T4/T3 results. The impact of these interferences varies between immunoassays, as assay formats and designs differ across manufacturers, resulting in different susceptibilities to matrix effects and binding perturbations.

Various forms of antibody interference exist and may cause falsely high or low FT4/FT3 concentrations.

## TSH assay interference

### Prevalence of TSH assay interference

Numerous causes of analytical interference in the TSH assay are reported in the scientific literature, including medications (biotin), genetic variation in *TSHB* (8), antibodies to components of the assay (anti-streptavidin or anti-ruthenium antibodies), autoantibodies (anti-TSH autoantibodies, also termed macro-TSH), and others (human anti-animal antibodies (HAMAs) or heterophilic antibodies) (6). Prevalence of such causes of artefactual results varies from 0.6–1.6% for macro-TSH to 0.4% for anti-reagent antibodies. The TSHβ variant is particularly prevalent (1%) in the South Asian population (4, 9).

### Overview of TSH assays

The advent of monoclonal antibodies allowed development of immunometric assay methodology (sandwich assay). This improved the specificity and drastically increased the functional sensitivity by dropping the limit of quantification from approximately 1 mIU/L with first-generation immunoassays to 0.01 mIU/L with third-generation immunoassays, which are now widely available (10). Depending on the manufacturer, the capture antibodies may be linked to magnetic bead covalently or with a system involving biotin/streptavidin affinity. The detection antibody is linked to the signal. TSH immunoassays are unlikely to be susceptible to the hook effect.

Unlike bioassays that measure the bioactivity of TSH, immunoassays cannot provide information on variations in glycosylation and bioactivity of TSH. Mass spectrometry TSH assays are currently not available for clinical use, although being developed. They may be able to distinguish isoform differences, but assay design and analytical sensitivity are still major issues (8).

The International Federation of Clinical Chemistry and Laboratory Medicine (IFCC) Committee for Standardization of Thyroid Function Tests (C-STFT) concluded that TSH cannot be fully standardised because no reference measurement procedure exists. Instead, the committee developed a harmonisation strategy using multi-assay comparisons with native, clinically relevant samples across multiple manufacturers (11). Although this approach improves agreement among commercial assays, it does not address immunoassay-related analytical interferences.

### Forms of TSH assay interference

#### Antibody interference

Antibodies are the main source of analytical interference in TSH immunoassays (Fig. 2). HAMAs and heterophilic antibodies, anti-streptavidin, or anti-ruthenium may cause under- or over-estimation of TSH concentration (12). Anti-TSH antibodies that are complexed with TSH are termed ‘macro-TSH’; this molecule is biologically inactive due to confinement to the intravascular compartment because of its high molecular size. None of the available immunoassays can discriminate all macro-TSH forms from bioactive free TSH, although some assays are less sensitive to its presence (e.g. Abbott assays, e.g. Alinity®) than others (e.g. Roche Cobas®) (6, 13, 14). Notably, transplacental transfer of maternal macro-TSH may result in biochemical findings that mimic transient congenital hypothyroidism in newborns, potentially leading to misdiagnosis and unnecessary treatment for congenital hypothyroidism (15).

#### Genetic

A single nucleotide change in *TSHB* encodes a variant TSHβ subunit that retains bioactivity but alters an epitope necessary to generate a TSH ‘sandwich’ in some (but not all) assays (8, 9).

#### Others

Non-specific interference has been observed in patients tested shortly after iodine-based contrast administration, causing under- or overestimation of results (16). Increasing use of therapeutic monoclonal antibodies may also raise the risk of antibody-related interference, although some studies report no such effects across multiple assays (17).

### Methods to identify TSH assay interference

**Figure 4:**
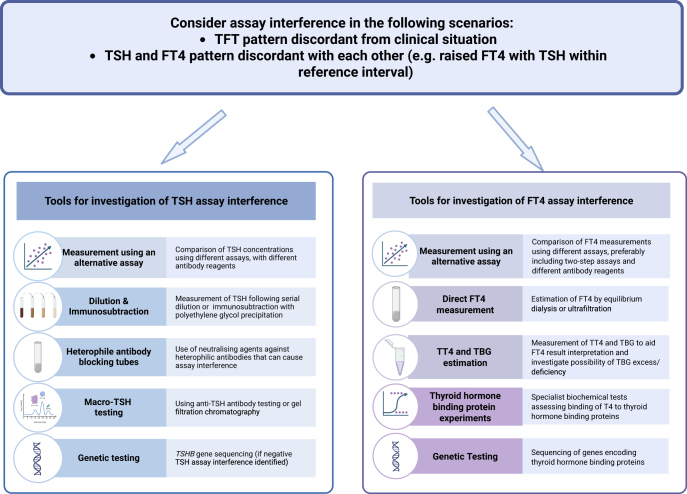
Summary of tools available to investigate suspected TSH and free T4 assay interference. Tools available to investigate TSH and free T4 assay interference are listed. These will not be available in the majority of laboratories, but are available in specialist referral laboratories. The tests performed will depend on the assay in which interference in suspected, the suspected cause of assay interference, and availability of testing in a given centre. Created in BioRender by Moran C (2026) https://BioRender.com/uv0w3ur.

The first and most simple strategy is the use of an alternative immunoassay employing antibodies with different epitopes and host species (6, 9). Depending on the suspected nature of interference, several other tests may be used (Fig. 4). Polyethylene glycol will differentially precipitate proteins based on solubility; in serum, immunoglobulins are readily precipitated (anti-reagent and anti-TSH antibodies). However, this test should be interpreted with caution as up to 70% of TSH immunoreactivity can be precipitated by polyethylene glycol in the absence of antibody interference; for this reason, platform and population specific cut-offs need to be established in each laboratory (18). Mixing studies can be carried out using sera with high TSH values; the presence of interference is likely to result in a non-linear reduction in TSH concentration. Heterophilic blocking tubes (HBTs) can be used to preabsorb interfering antibodies, before remeasuring TSH, but are relatively ineffective in most cases. More specialised methods are still available in some laboratories; these include gel filtration chromatography to identify macro-TSH and genetic testing (*TSHB*) when necessary (9). When the interference is thought to be related to a pharmaceutical product (e.g. biotin), the simplest method is to draw blood for TFT after cessation of the medication.

## T4 assay interference

Most laboratories routinely measure FT4 when assessing thyroid function, and most reports of T4 assay interference relate to FT4 assays. Therefore, the guidance here focuses primarily on FT4 measurement. Some information on total T4 (TT4) assay interference is provided separately below.

### Prevalence of FT4 assay interference

The prevalence of free T4 (FT4) assay interference varies by assay type. Single-step competitive immunoassays (widely used in routine laboratories) are more prone to interference than reference methods, such as equilibrium dialysis or ultrafiltration, which are typically confined to specialist centres. Inter-method variability in FT4 immunoassays exists, particularly when FT4 concentrations are high (19).

Genetic variants in THBPs also cause assay artefacts. Albumin variants occur in up to 1.8% of the population (7); transthyretinaemic hyperthyroxinaemia (TTH) has an estimated prevalence of 1:6,300 (7); and X-linked TBG variants occur in about 1:5,000 individuals, occasionally yielding falsely elevated results (9, 20).

Thyroid hormone autoantibodies (THAAs) are found in ∼1% of the general population (21) and up to 40% of patients with autoimmune thyroid disease (6, 22) but interfere in only 1–2% of affected samples (23, 24, 25). Although modern assays have reduced interference from heterophilic and anti-reagent antibodies, these remain unpredictable and continue to cause diagnostic confusion.

Monoclonal paraproteins may cause assay interference in up to 8% of people with discordant TFTs due to assay interference (26).

### Overview of FT4 assays

FT4 can be measured in clinical practice using two main approaches.

#### Indirect immunoassays

Using indirect methods of FT4 measurement, free hormone is inferred by competitive assay responses. These automated, high-throughput methods are widely used but are vulnerable to interference from autoantibodies, displacing agents, and genetic variation in THBPs. Because T4 is small, both current assay designs use single-antibody formats. In one-step assays, the sample, detection antibody, and competitive tracer are incubated together, whereas two-step assays add a wash step before tracer addition to limit interference from serum components.

#### Direct methods

In direct methods, bound T4 and FT4 are physically separated, using equilibrium dialysis (27) or ultrafiltration (28), and then, FT4 is measured using an immunoassay or LC–MS. These require more time, manual handling, and larger sample volumes, making them less suitable for routine use. However, they are more resistant to interference. The International Federation of Clinical Chemistry and Laboratory Medicine (IFCC) working group for standardisation of Thyroid Function Tests (WG-STFT) has established a reference measurement procedure for FT4 (equilibrium dialysis–isotope dilution–liquid chromatography/tandem mass spectrometry, ED–ID–LC/tandem MS) (29). Recently, alternative candidate reference methods for FT4 have been proposed (27, 30). None of these are readily available for routine clinical use.

### Forms of FT4 assay interference

#### Displacing agents

Some medications affect the accuracy of FT4 measurement, often due to displacement of T4 from THBPs (31). Heparin is the best described example: it stimulates lipoprotein lipase, generating free fatty acids *ex vivo* that displace T4 and falsely elevate FT4 (32). Markedly increased endogenous fatty acids can produce a similar effect. High doses of phenytoin and furosemide can also generate incorrect FT4 results if the assay design permits readjustment of the T4-binding equilibrium during measurement.

#### THBP variation

Although FT4 assays aim to minimise THBP dependence, many immunoassays are affected by genetic and physiological variation, including pregnancy (33). FDH, caused by albumin variants with increased T4 affinity (8), typically leads to FT4 overestimation in indirect assays, with the degree of interference being assay-specific (34). Direct methods are less affected but remain sensitive to assay conditions (35, 36). Interference from TTH is likewise assay-dependent and may fluctuate within individuals due to illness-related changes in TTR structure (8). TBG variants mainly affect TT4, but TBG deficiency can occasionally yield falsely high FT4 (9, 20).

#### Antibody interference

##### Thyroid hormone autoantibodies (THAAs)

THAAs can increase FT4 in one-step assays by binding the tracer, whereas two-step assays are less susceptible because THAAs are removed before tracer addition (6). Despite improved design of one-step methods, THAA-related interference (though rare) remains clinically significant.

##### Anti-reagent antibodies

Antibodies against assay components, including ruthenium, biotin, and streptavidin, may block tracer binding and produce spuriously high FT4 (12). Manufacturers incorporate blocking reagents, but complete prevention is not achievable (37).

#### Medications

Biotin supplements interfere with biotin–streptavidin-based competitive assays, typically causing falsely high FT4 (38). Chloral hydrate can cause erroneously raised TH concentrations (9).

#### Others

Markedly increased free fatty acid concentrations can raise FT4 artefactually (39), although concentrations required for interference are unlikely in patients with normal lipase activity (40). Clonal paraproteins may cause assay interference (26).

### Methods to investigate FT4 assay interference

Reviewing prescribed and over-the-counter medications that may alter T4 metabolism or displace T4 from binding proteins is a recommended first step before investigating assay interference. If displacement is suspected, TT4 measurement can help, as concentrations are expected to remain normal in euthyroid individuals without THBP variation. Serum protein electrophoresis and immunofixation may be helpful if a lymphoproliferative disorder is suspected (26). Direct methods, such as equilibrium dialysis, are resistant to most forms of interference, but require careful interpretation due to factors such as buffer composition, pH, and temperature (41). Notably, in cases of displacement, dialysis methods return a high FT4 concentration, since the FT4 concentration *in vitro* is genuinely elevated.

Given the cost, complexity, and limited availability of direct assays, repeating measurement using an alternative immunoassay can serve as a practical alternative, particularly since the performance characteristics of commonly used commercial assays are well established (6, 9). For laboratories using one-step assays, confirming results with a two-step method is advisable. Awareness of method bias is essential when comparing assays, especially as manufacturers continuously update immunoassays, potentially altering susceptibility to interference.

Although radioimmunoprecipitation for identifying anti-T3 and anti-T4 autoantibodies is rarely performed in clinical laboratories today, it remains a valuable tool (42). Specialist laboratories may perform radiolabelled T4-binding studies or electrophoretic techniques to assess abnormal T4–THBP interactions, although DNA sequencing now largely replaces older assays for detecting THBP variants (43). When inherited interference (e.g. FDH) is suspected, measuring THs in first-degree relatives can be a cost-effective and informative approach (Fig. 4).

### Total T4 assay interference

The IFCC WG-STFT uses isotope dilution–liquid chromatography/tandem mass spectrometry (ID–LC/tandem MS) as the reference measurement procedure for TT4 (44). TT4 measurement is strongly influenced by THBP variation and is therefore less reliable in settings such as pregnancy or critical illness. Its current use is largely confined to newborn screening programmes (45) and to evaluating suspected FT4 assay interference.

TT4 immunoassays remain the most common method of TT4 measurement, but can be affected by THAAs and heterophilic antibodies directed against the hormone or assay reagents, so TT4 measurement by mass spectrometry may be more accurate.

## T3 assay interference

### Prevalence of T3 assay interference

Total T3 or FT3 assay interference is less commonly reported than FT4 assay interference. While the exact prevalence is unknown, one study reported a prevalence of total T3 (TT3) assay interference of 1.2% of patients, with a higher frequency in females (1.4 vs 0.5%) (46).

### Overview of T3 assays

The assessment of TT3 and FT3 is technically more difficult than TT4 and FT4 measurements, due to their lower concentration in the serum (23). TT3 concentration is dependent on THBP and thus less reliable in conditions such as pregnancy and critical illness. In most laboratories, FT3 is measured by automated competitive immunoassays with a one-step or two-step architecture (22).

Indirect methods, such as free T3 index (FT3I) and T3/TBG ratio (47), are not frequently used and are not suitable for patients with THBP defects. Direct equilibrium dialysis and ultrafiltration coupled with RIA methods (48, 49) are no longer available in most laboratories.

The IFCC C-STFT has established a reference measurement procedure for FT3 (equilibrium dialysis–isotope dilution–mass spectrometry, ED–ID–MS) (50, 51) and TT3 (isotope dilution–liquid chromatography/tandem mass spectrometry, ID–LC/MS/MS) (44, 52, 53), but neither are widely available for routine testing.

### Forms of T3 assay interference

Similar to T4 assay interference, FT3/TT3 interference may occur due to antibody interference, medications, and THBP variations.

#### Displacing agents

Several drugs can displace THs from binding proteins that may increase both FT4 and FT3 serum concentrations, as described above (6).

#### THBP variation

Patients with FDH due to variants in codons 218 and 222 of the albumin gene have falsely raised FT3 and TT3 concentrations in many immunoassays, including one two-step platform (54). Isolated raised TT3 (but not TT4, FT4, or FT3) concentrations were described in a family harbouring a different (L66P) heterozygous mutation in *ALB* (55).

#### Antibody interference

Antibodies are the main source of analytical interference in TT3/FT3 immunoassay (6). HAMAs and heterophilic antibodies ((56, 57), new), anti-T3 autoantibodies (24), anti-streptavidin (12), anti-ruthenium (58), paraproteins (59), and rheumatoid factor (60) can cause overestimation of TT3 and/or FT3 concentrations. The frequency of interference with these antibodies is low, variable among different assays, and the relationship between antibody concentrations and severity of interference is not linear (24, 25).

#### Medications

Therapeutic TH analogues, such as triiodothyroacetic acid (TRIAC) (61) and diiodothyropropionic acid (DITPA) (62), cross-react to variable extents on different FT3 and TT3 assays. Biotin treatment is associated with spuriously high FT3 (63) with a mechanism similar to anti-streptavidin antibodies. The sedative agent chloral hydrate may cause falsely elevated FT3 (9).

#### Others

A pre-analytical cause of spuriously increased TT3 due to surfactant agents in blood collection tubes has been reported (64).

### Methods to investigate T3 assay interference

To investigate possible TT3/FT3 interference, the initial and most simple strategy is the use of an alternative one-step, or ideally two-step, immunoassay (6) or, if available, an alternative method such as LC-MS/MS. Remeasurement of both TT3 and FT3 can be helpful, since discrepant results are suggestive of interference. In one study of 120 samples with spuriously increased FT4, 71 patients had raised FT3 too, but TT3 concentrations were normal in more than 95% (65). HBTs can be useful, but the results can be assay-dependent and false positives may occur (66). PEG precipitation has been used in suspected FT3 interference due to paraproteins, but dilution of the sample changes the ratio of bound to unbound T3 (67), so it is advisable to compare the results with those of controls and genuine hyperthyroid patients (68).

## Interference in other assays used in the assessment of thyroid disease

### Thyroglobulin assay interference

#### Prevalence of thyroglobulin assay interference

Measurement of thyroglobulin (Tg) is primarily used to identify residual thyroid tissue or recurrence/metastases from a previously treated differentiated thyroid cancer (69, 70). The most common type of interference in the immunoassays is the presence of thyroglobulin antibodies (TgAbs) (71). The prevalence of these antibodies is high in normal individuals (in females up to 15–20%) (70) and also in patients with thyroid cancer (25%), tending to disappear after total thyroidectomy (72, 73) and remission (70, 74).

Other causes of Tg assay interference are likely only present in 1–2% of measurements, but considering the large volume of Tg measurements globally, and the emphasis placed on these results in cancer follow-up, this results in a large burden for healthcare systems and patients (70).

#### Overview of thyroglobulin assays

Tg is measured by an immunometric assay, which can be susceptible to interference (69, 70, 75). More recently, measurement by liquid chromatography–tandem mass spectrometry (LC–MS/MS) assay has been introduced (76), but these are not universally available due to the cost and low sensitivity, despite lesser influence from autoantibodies (77). In general, the variability between assays is very high, even after introduction of an international reference calibrator CRM457 (78), which reduced the inter-assay variability from approximately 60 to 30% (79, 80).

#### Forms of thyroglobulin assay interference

As stated above, the most common type of interference in the immunoassays is the presence of thyroglobulin antibodies (TgAbs). Tg assay interference may also be due to anti-thyroid hormone antibodies, heterophilic antibodies, anti-ruthenium and anti-streptavidin antibodies (74), rheumatoid factor (81), and biotin (63). It is also likely that other immunoglobulins, autoantibodies, and proteins interfere in Tg assays; for example, therapeutic infusions of intravenous immunoglobulin may contain TgAbs. A high-dose hook effect has been described in Tg measurements (82).

#### Methods to investigate thyroglobulin assay interference

TgAb measurement is recommended for all samples submitted for thyroglobulin (Tg) testing to help identify potential assay interference. However, TgAb assays are variable: antibodies may be present but undetected or detected without affecting the Tg assay. Heterophilic antibodies pose a greater challenge and may require specific approaches, including PEG precipitation, antigen blocking, or Tg measurement using an alternative assay. LC-MS/MS can be used when Tg assay interference is suspected, although falsely low Tg concentrations have been reported in some patients with structural disease and positive TgAbs (76).

### Calcitonin assay interference

#### Prevalence of calcitonin assay interference

Calcitonin (CT) assays are important diagnostic tools primarily used for the detection and monitoring of medullary thyroid carcinoma. Although less frequent, interference has been reported in CT assays (83).

#### Overview of calcitonin assays

These assays typically employ two-site immunoassay techniques, with chemiluminescence signalling. The limit of quantification of CT immunoassays has been decreasing over the years, resulting in increased sensitivity.

#### Forms of calcitonin assay interference

The presence of heterophilic antibodies can lead to falsely elevated CT concentrations (84), as can autoantibodies that cross-react with the assay components. For example, in one case, calcitonin measurements were confounded by the presence of rheumatoid factor, leading to significantly elevated calcitonin concentrations despite the absence of medullary thyroid carcinoma (81). Macro-calcitonin interferes with CT assays by mimicking elevated CT concentrations without actual increases in the hormone (85). Another issue is the high-dose hook effect, where extremely high analyte concentrations saturate the assay’s detection antibodies, resulting in falsely low readings (83).

#### Methods to investigate calcitonin assay interference

CT interference should be considered if the result is at variance with the clinical picture. Heterophilic blocking tubes, dilution studies, or CT measurement using alternative assays could be employed to investigate CT assay interference.

### Anti-receptor antibody (TRAb) assay interference

#### Prevalence of TRAb assay interference

The specificity and sensitivity of anti-TSH receptor autoantibodies (TRAbs) for Graves’ disease are excellent such that a positive TRAb result is almost diagnostic for Graves’ disease (86). However, as TRAb methods are based on immunoassay techniques, they are likely to suffer the same interferences as described above, although the prevalence has not been determined.

#### Overview of TRAb assays

In clinical practice, most TRAb assays are based on the displacement of a labelled monoclonal anti-TSH receptor antibody from immobilised recombinant TSH receptors.

As both blocking and stimulating TRAbs exist (87), a bioassay is required to discriminate between these species. Recent development of rapid and robust TRAb bioassays may enable clinical laboratories to distinguish between blocking and stimulating TRAbs more readily (88, 89).

#### Forms of TRAb assay interference

Biotin (90) and anti-streptavidin antibodies (91) have been described to cause TRAb interference, resulting in falsely elevated TRAbs.

#### Methods to investigate TRAb assay interference

TRAb interference should be considered if the result is at variance with the clinical picture or TH concentrations. Repeat testing following biotin withdrawal, or use of an alternative assay (or bioassay), if available, is advised.

### Anti-thyroglobulin antibody (TgAb) assay interference

#### Prevalence of TgAb assay interference

The prevalence of anti-thyroglobulin antibodies (TgAbs) is high in most populations, as mentioned above. The prevalence of TgAb assay interference is not known.

#### Overview of TgAb assays

TgAb measurement is cumbersome, with high inter-method variability and wide variation in limits of detection and reference intervals (70, 92).

#### Forms of TgAb assay interference

Very high concentrations of thyroglobulin in serum can interfere with the autoantibody measurement in some assays (93). Interference from thyroglobulin is assumed to vary with the initial concentration, i.e. low TgAb concentrations only need mildly elevated Tg to interfere with the measurement, while very high TgAb concentrations need very high Tg concentrations to interfere with the quantification (94, 95). It is also likely that other immunoglobulins (including IvIg treatment), autoantibodies and proteins may interfere in TgAb assays (96).

#### Methods to investigate TgAb assay interference

TgAb interference should be considered if the result is at variance with the clinical picture. Repeat testing using an alternative method could be considered.

### Anti-TPO antibody (TPO-Ab) assay interference

#### Prevalence of TPO-Ab assay interference

TPO-Abs are mainly used for the diagnosis of autoimmune thyroid disease but are often used as markers of autoimmunity in general (96, 97). The prevalence of TPO-Ab assay interference is not known.

#### Overview of TPO-Ab assays

TPO-Ab assays used in Europe are mainly third-generation automated immunoassay platforms (98). The upper reference limit is method-dependent and often arbitrarily established, in part due to uncertainty of criteria used to define the reference population (99). The sensitivity and specificity of TPO-Abs for autoimmune thyroid disease are relatively poor (97).

#### Forms of TPO-Ab assay interference

TPO-Ab immunoassays are vulnerable to interference from exogenous antibodies (e.g. intravenous immunoglobulin) and endogenous factors, such as heterophilic antibodies and rheumatoid factor (91). In addition, structural homology between thyroid peroxidase and myeloperoxidase permits antibody cross-reactivity (100), particularly in denatured or solid-phase assay conditions, although this effect appears limited in native systems (101). These mechanisms should be considered when laboratory findings are inconsistent with the clinical picture.

#### Methods to investigate TPO-Ab assay interference

If TPO-Ab assay interference is suspected, repeat testing using an alternative method, or measurement of other thyroid autoantibodies, could be considered.

## Information on investigation of assay interference for specialists in laboratory medicine

Numerous laboratory methods are available to detect and investigate interference. Table 2 outlines these, providing a valuable resource for specialists in laboratory medicine.

**Table 2:** Comprehensive overview of detection strategies for assay interference. Summary of laboratory methods used to identify and investigate assay interference in TSH, T4, thyroglobulin, and thyroid autoantibody assays.

Method	Detection mechanism	Effectiveness	Notes	Potential limits	References
Dilution testing	Serial dilution of sample	Effective for identifying non-linearity	Helps to confirm the presence of macro-analytes or interference by revealing non-linearity	Will not identify the nature of the interference	(13, 107)
Remeasurement using an alternative assay	Comparing analyte measurements using one or more alternative assays	Effective for confirmation of interference	Discrepancies between different methods can indicate interference	Requires access to multiple platforms; cost and time-intensive	(5, 108)
Polyethylene glycol treatment	Precipitation with polyethylene glycol	Good for detecting macro-aggregates	Useful to differentiate true analyte from bound forms causing interference	Does not eliminate all forms of interference	(109, 110)
Pre-treatment	Use of heterophilic blocking tubes	Highly effective for heterophilic antibody interference	Directly targets and neutralises interfering antibodies prior to analysis	Limited to known heterophilic interactions	(56, 111)
Preventing biotin interference	Biotin supplementation awareness and assay design	Highly effective	Advising patients to withhold biotin supplements before testing and using assays not affected by biotin	Patient compliance needed; not all assays may be biotin-free	(6, 112)
Antibody neutralisation	Use of specific neutralising agents against known interferences	Varies with the target interference	Employed to directly tackle known interferences such as biotin, rheumatoid factor, or animal antibodies	Specificity required; may not work for unidentified interferences	(6)

Dilution testing is a straightforward approach to suspected TSH assay interference, where serial dilution reveals non-linearity, indicative of macro-analytes or other interferences. Although effective, it does not pinpoint the exact nature of the interference, making it a preliminary rather than conclusive test, and requiring adequate dilution controls.

Analysing the same sample using alternative immunoassays can help identify assay interference, particularly when discrepant results are obtained across assays. This process requires access to multiple platforms and can be both cost- and time-intensive but is effective in confirmation.

Polyethylene glycol (PEG) treatment helps differentiate ‘true’ analytes from bound forms causing interference by precipitating macro-aggregates. While good for detecting certain types of interference, it cannot address all forms.

Pre-treatment with heterophilic blocking tubes offers a targeted approach to neutralise heterophilic antibody interference before analysis. This method is effective but limited to known heterophilic interactions; these reagents are usually already incorporated in modern immunoassays.

Finally, preventing biotin interference involves pre-treatment solution or advising patients to withhold biotin supplements before testing and using assays designed to be unaffected by biotin. Over time, assay redesign by manufacturers can reduce assay susceptibility to specific forms of interference. Together, these methods form a comprehensive toolkit for specialists in laboratory medicine to address and manage assay interferences, ensuring that TFT results are both accurate and reliable in reflecting the thyroid status of patients.

Access to these methods and level of expertise in detecting and investigating assay interference will vary between laboratories.

## Investigation, management, and follow-up of people in whom interference is identified

Detection and confirmation of assay interference is important; in patients without thyroid disease, it allows the physician to clearly avoid unnecessary further investigation and treatment, and in patients with thyroid disease (for example, a patient with Hashimoto’s thyroiditis with FT4 assay interference), it facilitates appropriate interpretation of thyroid function tests. In those with genuinely discordant thyroid function (such as TSH-secreting pituitary tumours or resistance to thyroid hormone β), assay interference should always be excluded prior to progressing to further, specialised, investigations, as suggested in specific ETA guidelines (102, 103).

In patients with evidence of FT4 assay interference, TSH alone can be used to guide thyroid status. If an inherited cause of assay interference is identified, screening should be offered to first-degree family members. For individuals with confirmed TSH assay interference, an alternative assay that accurately measures TSH may be used to evaluate thyroid function. However, in certain cases of TSH interference, such as macro-TSH, obtaining a reliable TSH measurement may not be feasible. In these situations, thyroid assessment relies solely on clinical context along with FT4 and FT3 values.

If assay interference has been identified, clear documentation should be provided to the patient and primary care practitioner and recorded in the patient’s medical record. The concept of assay interference can be difficult to explain to the patient, so access to high-quality, reliable patient information from national or international patient organisations would be valuable. The accompanying plain language summary of this guideline (Appendix 1) is intended to provide an accessible overview of the key messages and recommendations contained in this guideline for non-specialist readers, including patients, carers, healthcare professionals outside the speciality, and the wider public.

Notably, as assays change and evolve, assay interference may not be present indefinitely in an individual, so patients need to be re-assessed by an endocrinologist if clinical features of thyroid disease emerge in future.

## Conclusion and future directions

As we look to the future of thyroid function tests (TFTs), a convergence of emerging technologies stands poised to significantly reduce assay interferences and enhance diagnostic accuracy. Advances in omics technologies and automated mass spectrometry (MS) are at the forefront, promising to deepen our understanding of thyroid biochemistry and pathophysiology (76). Automated MS, in particular, may offer a robust solution to the prevalent issue of antibody interferences in traditional immunoassays, by providing a more specific and reliable means of measuring thyroid markers. Concurrently, the rise of artificial intelligence (AI) is set to transform TFTs through the *in silico* design of assays (104). By employing machine learning algorithms, these AI-enhanced designs can anticipate and circumvent potential interferences before the assays are even deployed in a clinical setting. Furthermore, integrating these AI models with electronic medical records enables the analysis of large datasets to detect patterns that predict likelihood of interference, thereby improving the predictability and reliability of thyroid testing. Integration of AI not only aims to refine the accuracy of TFTs but also facilitates a shift towards personalised medicine. By tailoring TFTs to the individual biochemical profiles of patients, these technologies hold the potential to greatly improve disease management and patient outcomes (105). Together, these advancements herald a new era in endocrinology, where technology-driven insights and innovations lead to more precise, interference-resistant thyroid function testing. However, the adoption of emerging technologies should be rigorously validated and ethically managed to ensure that they meet high standards of effectiveness and fairness (106).

## Declaration of interest

The task force had no commercial support, and the task force members declare no conflicts of interest.

## Funding

UFR’s research salary was funded by Kirsten and Freddy Johansen’s Fund.

## Appendix 1

### Understanding the 2026 ETA Guideline on Interference in Immunoassay Measurements Used in the Assessment of Thyroid Function: A Plain Language Summary

Thyroid blood tests are commonly used to diagnose and monitor thyroid conditions, including an underactive thyroid (hypothyroidism), an overactive thyroid (hyperthyroidism) and thyroid cancer. These tests usually provide reliable information and are essential for guiding treatment decisions.

Most thyroid tests are performed using laboratory methods called **immunoassays**, which measure hormones, such as thyroid stimulating hormone (TSH), thyroxine (T4), triiodothyronine (T3), and thyroid-related antibodies, in blood samples. Although these tests are generally accurate, results can occasionally be misleading because of **assay interference**. Assay interference occurs when substances in the blood, inherited biological differences, or medications affect how the laboratory test performs, leading to results that do not accurately reflect thyroid gland function.

Incorrect thyroid test results may lead to delayed diagnosis, unnecessary investigations, inappropriate treatment, or confusion for patients and healthcare professionals. In some cases, misleading test results may resemble rare thyroid disorders, making interpretation particularly challenging.

This guideline summarises the causes of assay interference in thyroid-related laboratory testing and provides recommendations for healthcare professionals investigating unexpected or conflicting thyroid test results.

The guideline highlights that interference may arise from several sources. These include naturally occurring antibodies that affect laboratory measurements, medicines and some supplements (particularly biotin, commonly used in hair and nail supplements), and inherited differences in proteins that transport thyroid hormones in the bloodstream. Pregnancy, severe illness, and some medical treatments may also influence the interpretation of thyroid test results.

The susceptibility to interference varies between different laboratory methods. Some machines used for thyroid hormone levels are more resistant to interference than others, meaning that the same blood sample may occasionally produce different results depending on the method used. For this reason, unexpected or unusual thyroid blood tests should be interpreted carefully and always in the context of a person's symptoms, examination findings, medications, and medical history.

A major recommendation of the guideline is that healthcare professionals should consider assay interference when thyroid blood test results do not fit the clinical picture.

The guideline also emphasises the importance of collaboration between clinicians and laboratory medicine specialists when interference is suspected. Investigation may involve repeating tests using an alternative laboratory method, reviewing medications and supplements, or performing more specialised laboratory analyses. In selected cases, referral to specialist centres with expertise in complex thyroid function testing may be required.

The guideline also discusses interference affecting other thyroid-related tests, including thyroglobulin, calcitonin, and thyroid antibody measurements, which are commonly used in thyroid cancer and autoimmune thyroid disease. Awareness of possible interference in these tests is important because inaccurate results may influence diagnosis, treatment decisions, and long-term follow-up.

Importantly, the guideline does not suggest that thyroid blood tests are unreliable. On the contrary, thyroid testing is highly accurate for most people. However, recognising the small number of situations in which results may be misleading is important to avoid unnecessary investigations or inappropriate treatment.

Advances in laboratory technology, including improved assay design and more sophisticated methods of thyroid hormone measurement, are expected to reduce interference in the future and improve test accuracy further. Increased awareness among healthcare professionals and close communication between clinical and laboratory teams remain central to ensuring thyroid test results are interpreted correctly.

This guideline provides practical recommendations to help healthcare professionals recognise, investigate, and manage assay interference, with the aim of improving the safety, reliability, and interpretation of thyroid function testing.
